# Prediction of lymph node metastasis in papillary thyroid carcinoma using non-contrast CT-based radiomics and deep learning with thyroid lobe segmentation: A dual-center study

**DOI:** 10.1016/j.ejro.2025.100639

**Published:** 2025-02-24

**Authors:** Hao Wang, Xuan Wang, Yusheng Du, You Wang, Zhuojie Bai, Di Wu, Wuliang Tang, Hanling Zeng, Jing Tao, Jian He

**Affiliations:** a Department of Radiology, The Fourth Affiliated Hospital of Nanjing Medical University, Nanjing 210031, PR China; b Department of Radiology, Zhongda Hospital Southeast University (JiangBei), Nanjing 210048, PR China; c Department of General Surgery, The Fourth Affiliated Hospital of Nanjing Medical University, Nanjing 210031, PR China; d Department of Nuclear Medicine, Nanjing Drum Tower Hospital, The Affiliated Hospital of Medicine school, Nanjing University, Nanjing 210008, PR China

**Keywords:** Lymph node metastasis, Papillary thyroid cancer, Deep transfer learning, Radiomics, Non-contrast CT

## Abstract

**Objectives:**

This study aimed to develop a predictive model for lymph node metastasis (LNM) in papillary thyroid carcinoma (PTC) patients by deep learning radiomic (DLRad) and clinical features.

**Methods:**

This study included 271 thyroid lobes from 228 PTC patients who underwent preoperative neck non-contrast CT at Center 1 (May 2021–April 2024). LNM status was confirmed via postoperative pathology, with each thyroid lobe labeled accordingly. The cohort was divided into training (n = 189) and validation (n = 82) cohorts, with additional temporal (n = 59 lobes, Center 1, May–August 2024) and external (n = 66 lobes, Center 2) test cohorts. Thyroid lobes were manually segmented from the isthmus midline, ensuring interobserver consistency (ICC ≥ 0.8). Deep learning and radiomics features were selected using LASSO algorithms to compute DLRad scores. Logistic regression identified independent predictors, forming DLRad, clinical, and combined models. Model performance was evaluated using AUC, calibration, decision curves, and the DeLong test, compared against radiologists' assessments.

**Results:**

Independent predictors of LNM included age, gender, multiple nodules, tumor size group, and DLRad. The combined model demonstrated superior diagnostic performance with AUCs of 0.830 (training), 0.799 (validation), 0.819 (temporal test), and 0.756 (external test), outperforming the DLRad model (AUCs: 0.786, 0.730, 0.753, 0.642), clinical model (AUCs: 0.723, 0.745, 0.671, 0.660), and radiologist evaluations (AUCs: 0.529, 0.606, 0.620, 0.503). It also achieved the lowest Brier scores (0.167, 0.184, 0.175, 0.201) and the highest net benefit in decision-curve analysis at threshold probabilities > 20 %.

**Conclusions:**

The combined model integrating DLRad and clinical features exhibits good performance in predicting LNM in PTC patients.

## 1 Introduction

The incidence of thyroid cancer has risen markedly in recent years, ranking as the third most common malignancy in China, with a notably higher incidence rate among females than males [1]. Papillary thyroid carcinoma (PTC) represents the most prevalent histological subtype of thyroid cancer, accounting for 80 %-90 % of cases [2], [3]. While most PTC cases are considered indolent [4], certain aggressive subtypes present a heightened risk of lymph node metastasis (LNM) and postoperative recurrence [5], [6], [7], [8]. Current primary treatment options for PTC include total or near-total thyroidectomy and ultrasound (US)-guided microwave ablation (MWA) [9]. However, surgical intervention carries risks of complications, such as recurrent laryngeal nerve damage and unintentional parathyroid removal [10], which can significantly impact the patient’s quality of life. Additionally, MWA may not be suitable for patients with intermediate- to high-risk PTC exhibiting LNM [11]. Consequently, preoperative identification of LNM is essential for guiding treatment choices [12], as precise LNM assessment enables clinicians to make targeted therapeutic decisions with substantial clinical impact.

According to the National Comprehensive Cancer Network (NCCN) guidelines for thyroid cancer, preoperative imaging is advised for LNM detection in patients with PTC, informing surgical decision-making [13]. US remains the preferred imaging modality due to its non-radiative, non-invasive, and convenient characteristics [14]. However, research indicates limited diagnostic for smaller lymph nodes, such as those in the central compartment (sensitivity 65.6 %, specificity 59.6 %), and challenges in visualizing deeper regions (e.g., retropharyngeal space and upper mediastinum), affecting diagnostic accuracy (69.79 %) [15]. Contrast-enhanced computed tomography (CECT) is effective in evaluating PTC size and extent, making it a common tool in preoperative assessments. Nonetheless, its diagnostic capabilities predominantly rely on morphological characteristics and lack sufficient quantitative parameters. A comparative study of CECT versus US was conducted, with the results indicating that CT exhibited lower sensitivity, specificity and accuracy (37.1 %, 94.8 %, 71.4 %) in comparison to US (49.8 %, 92.1 %, 75.0 %) [16]. Moreover, the iodinated contrast agents used in CECT can induce hyperthyroidism and delay radioactive iodine (I-131) treatment [17], [18], [19]. These imaging techniques are also unsuitable for patients with iodine allergies or renal impairment. Therefore, the development of a convenient, minimally burdensome method for diagnosing LNM holds considerable clinical value.

Radiomics facilitates the quantitative extraction of diverse features from medical images [20], [21], capturing nuanced tumor characteristics often undetectable through conventional imaging techniques [22]. Deep learning, particularly through deep convolutional neural networks, identifies intricate patterns and structures, allowing for the extraction of high-dimensional representative features [23]. Deep transfer learning (DTL), which utilizes pre-trained neural networks with fine-tuned parameters on new datasets, enhances model performance while optimizing resource use [24]. The integration of radiomics and DTL has shown encouraging results in differentiating benign from malignant thyroid nodules [25], predicting LNM [26], and anticipating immunohistochemical outcomes [27]. The combination of radiomics and DTL offers substantial potential for advancing personalized precision treatment [28]. While existing studies on LNM prediction in patients with PTC have primarily utilized US and CECT images, the predictive value of non-contrast CT (NCCT) images remains unexplored; Conventional LNM prediction studies are challenging to accurately match pathological findings due to the complexity of the cervical lymph nodes and the lower sensitivity of imaging, especially in the case of multiple nodules where manual localisation of the nodules and definition of the nodules margins may be biased. This study used lobe thyroid tissue segmentation with the aims to develop a model based on preoperative CT images that integrates radiomic, DTL features, and clinical variables to predict LNM risk in patients with PTC, providing valuable support for clinicians in treatment planning.

## 2 Materials and methods

### 2.1 Patients

**Fig. 1:**
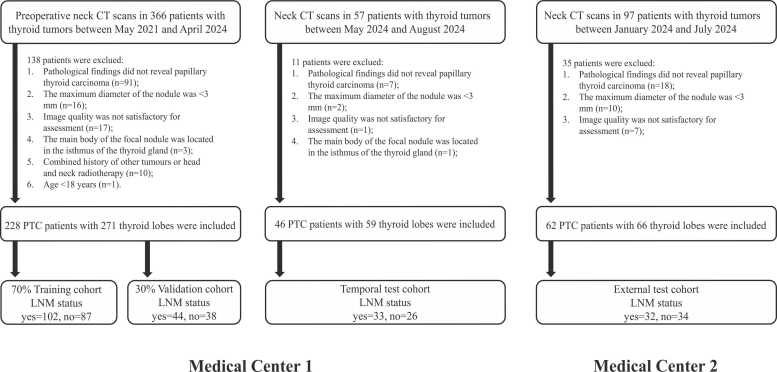
Flowchart of study inclusion. LNM: lymph node metastasis.

This study complies with the principles outlined in the Declaration of Helsinki and received approval from the Ethics Committee of the Fourth Affiliated Hospital of Nanjing Medical University (approval number: 20240628-K077), with informed consent requirements waived for all participants. Patients who underwent thyroidectomy and received a pathological diagnosis of PTC at the Fourth Affiliated Hospital of Nanjing Medical University between May 1, 2021, and April 30, 2024, were retrospectively included. Inclusion criteria were as follows: (1) postoperative pathological confirmation of PTC, (2) pathological findings from cervical lymph node dissection, and (3) preoperative cervical CT conducted within 15 days prior to surgery. Exclusion criteria were: (1) nodule diameter less than 3 mm, (2) poor image quality with substantial breathing or beam hardening artifacts, (3) PTC tumor primarily located in the thyroid isthmus or pyramidal lobe, (4) history of other malignancies or head and neck radiotherapy, and (5) age under 18 years. After excluding 138 patients who did not meet eligibility criteria, 228 patients with PTC were included in the study. Unilateral thyroid lobes were categorized by location relative to the thyroid isthmus, yielding 271 unilateral lobes with pathological outcomes. LNM status was determined at the patient level through postoperative pathological examination. For bilateral PTC cases, both thyroid lobes were segmented and labeled identically based on the patient’s overall LNM status. Thyroid lobes were classified as LNM-positive or LNM-negative based on lymph node pathology findings and then randomly allocated to training and validation cohorts in a 7:3 ratio. Additional retrospective data from May 1, 2024, to August 31, 2024, and from Zhongda Hospital, Southeast University (January 1, 2024, to June 30, 2024), were also included. After excluding 11 and 35 patients, respectively, according to the same criteria, 59 lobes (from 46 patients) were incorporated into the temporal test cohort, and 66 lobes (from 62 patients) formed the external test cohort. The study adhered to the Checklist for Evaluating Radiomics Reporting (CLEAR list) to ensure the accuracy and rigor of the radiomics and deep learning processes [29], with the study design and workflow illustrated in Fig. 1.

### 2.2 Imaging acquisition

**Fig. 2:**
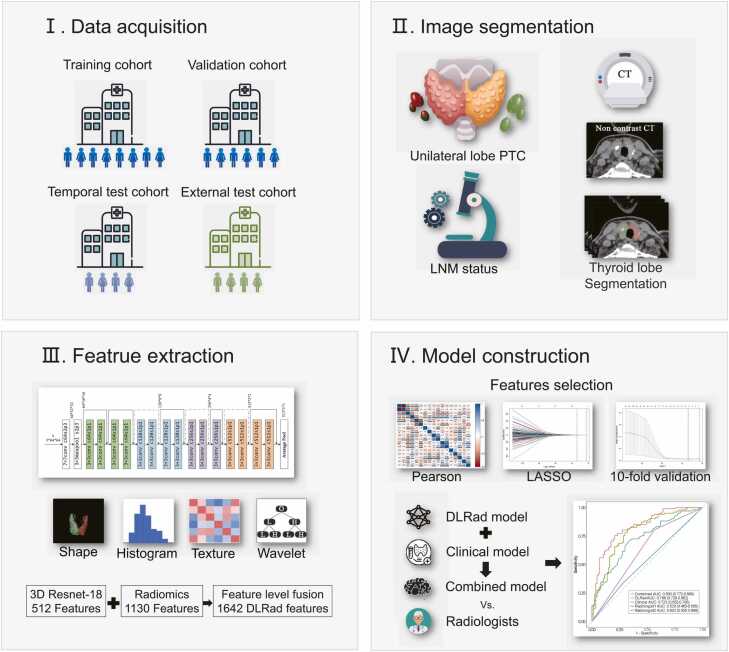
Workflow of model development. DLRad score: deep learning radiomics score; PTC: papillary thyroid cancer; LNM: lymph node metastasis.

In this study, patients in the training cohort, validation cohort, and temporal test cohort underwent neck NCCT and CECT scanning using the IQon Spectral CT (Philips Medical Systems, The Netherlands) while positioned head-first and supine. To ensure optimal image quality, patients were instructed to breathe calmly while avoiding swallowing, with the jaw elevated and shoulders stretched downward to minimize overlap in the neck region. Scans extended from the base of the skull to the superior mediastinum. Scanning parameters included a tube voltage of 120 kVp, with an automatically adjusted tube current *via* the DoseRight technique (index 23), yielding an average of 145 mAs, a collimator width of 64 × 0.625 mm, and a field of view of 300 mm. The pitch was set to 0.969, and images were captured with a 512 × 512 matrix, with a 1 mm layer thickness and spacing. Iterative reconstruction was implemented using iDose level 4 technology, and the window width was 350 Hu, and the window level was 60 Hu [30]. For CECT, scans were acquired at 25 and 60 seconds post-contrast injection. All images were saved in DICOM format within the PACS system. Only NCCT images were used in this analysis. Fig. 2

For the external test cohort, patients underwent neck NCCT scan using the Revolution CT (GE Healthcare, USA). Scanning parameters included a tube voltage of 120 kVp, automatic tube current adjustment via mAs, a collimator width of 256 × 0.625 mm, a field of view of 250 mm, a pitch of 0.992, and a 512 × 512 matrix for image capture. Images were reconstructed using the ASIR 50 % algorithm, with a slice thickness and spacing of 0.625 mm. The window settings were a width of 350 Hu and a level of 50 Hu.

### 2.3 Pathological results, imaging features, and clinical variable collection

All pathological analyses were conducted on thyroid and lymph node specimens that had been surgically excised, formalin-fixed, and paraffin-embedded. Two experienced pathologists (with 10 and 15 years of experience in thyroid pathology, respectively) independently confirmed the presence of PTC and identified LNM based on histological evaluation, ensuring diagnostic consistency. Any discrepancies were resolved through consensus discussion. Imaging features—including tumor size group (small: ≤5 mm, medium: 5–10 mm, large: >10 mm), presence of multiple nodules, calcification, and cystic degeneration—were assessed by radiologists A and B (with 15 and 25 years of diagnostic experience, respectively) using a PACS workstation. In instances of disagreement, a third radiologist with 25 years of experience was consulted to reach a consensus. Out of 396 thyroid lobes evaluated, only 12 cases (3.03 %) required consensus review, primarily due to minor measurement variability in tumor size or challenges in identifying small nodules. Calcification and cystic degeneration showed virtually no disagreements, reflecting the clarity of these diagnostic criteria. Baseline clinical data, including variables such as age, gender, BMI, and laboratory markers (free triiodothyronine [FT3], free thyroxine [FT4], thyroid-stimulating hormone [TSH], thyroglobulin antibody [TGAb], and thyroid peroxidase antibody [TPOAb]), were retrieved from the hospital information system (HIS).

Additionally, radiologists A and B participated in a double-blind diagnostic assessment of LNM status for all thyroid lobes based on the NCCT in training cohort and validation cohort to facilitate human-machine comparison. To ensure objectivity, the double-blind LNM assessment was performed one month after the qualitative analysis, following a standardized training session on the diagnostic criteria. The diagnostic criteria for LNM are as follows: (1) Lymph node volume increases significantly, (2) Lymph node aspect ratio is close to 1 and morphology tends to be round, (3) Lymph node density increases or internal density is uneven, (4) Multiple lymph nodes adhere to each other, forming a tendency to fusion.

### 2.4 Lobe thyroid tissue segmentation

Manual segmentation was performed using 3D Slicer version 5.7.0 (https://www.slicer.org). Due to the small volume of thyroid tissue, image resolution was enhanced by resampling to 0.5 mm × 0.5 mm × 1 mm. An experienced radiographer A (the first author), with 14 years of professional experience in CT imaging, manually delineated the region of interest (ROI) on axial CT images, guided by postoperative pathological findings, including PTC tumor location (left and/or right lobe) and LNM status. The thyroid gland was divided into left and right lobes, with the isthmus serving as the central dividing region. Radiographer A then proceeded in layers along the thyroid boundaries to ensure accurate segmentation. All ROIs were reviewed and validated by Radiologist A to ensure precision.

### 2.5 Deep learning and radiomics feature extraction

In the Python 3.7-based PyTorch framework, a 3D ResNet-18 deep convolutional network was utilized as the deep transfer learning model, from which 512 DTL features were extracted from the average pooling layer. Radiomic features were generated using the Pyradiomics package (https://github.com/Radiomics/pyradiomics), yielding a total of 1130 features. This dataset included 14 shape features, 18 first-order histogram features, 75 high-order texture features (covering GLCM, GLDM, GLRLM, GLSZM, and NGTDM), 297 Laplacian of Gaussian (LOG) features, and 726 wavelet features.

### 2.6 Consistency validation analysis

To ensure reproducibility in manual segmentation and mitigate subjectivity, 40 thyroid lobes were randomly selected, with Radiologist A re-segmenting the ROI for DTL and radiomic feature extraction. Radiographer A then repeated segmentation on the same thyroid lobes one week later to extract identical features. Intraclass and interclass correlation coefficients (ICCs) were applied to evaluate feature extraction consistency, excluding features with an ICC < 0.8.

### 2.7 Feature fusion and preprocessing

To enhance complementarity among features of varying dimensions and reduce overfitting risk, a feature-level fusion strategy was implemented, merging DTL and radiomic features into a deep learning radiomics (DLRad) dataset. All features underwent Z-score normalization to standardize magnitudes, calculated as follows: Z-score = (X - μ) / σ, where X represents the feature value, μ the mean, and σ the standard deviation.

### 2.8 Establishment of DLRad score

Feature selection commenced with correlation filtering, retaining only one feature from pairs with Pearson correlation coefficients > 0.7 to minimize multicollinearity. Subsequently, LASSO (Least Absolute Shrinkage and Selection Operator) was employed, using 10-fold cross-validation to identify the optimal λ penalty coefficient, thereby retaining non-zero features. The DLRad score for each thyroid lobes was computed as follows: DLRad score = Σ(feature value * feature weight) + intercept. To determine whether feature weights predominantly derive from the PTC tumors itself or surrounding tissues and to evaluate potential false positives from benign thyroid lobes, the study included 37 pairs of unilateral thyroid lobe containing PTC with contralateral nodular goitre and thyroid adenoma (benign conditions) from both the training and validation cohort. Differences in features and DLRad scores between benign and malignant lobes were analyzed, providing insights into the contribution of different tissue regions to the diagnostic model and the risk of false positives in benign lobes.

### 2.9 Model construction and evaluation

Univariate and multivariate logistic regression analyses were conducted on clinical variables, imaging features, and laboratory indicators to identify relevant features, followed by the construction of a clinical model using the retained features. A multivariate logistic regression algorithm was applied to build a deep learning radiomics model based on DLRad scores, which was subsequently combined with the clinical model to create an integrated model. The area under the curve (AUC) of receiver operating characteristic (ROC) curves was used to assess each dataset and select the optimal model, which was represented visually with a nomogram. AUC differences were analyzed using the DeLong test, while calibration and decision curve analysis (DCA) provided further evaluation of each model’s diagnostic performance. Moreover, in order to validate the stability of tumor features of differing sizes in the lateral lobes of the thyroid, this study evaluated lobes of different tumor size subgroups in both the training cohort and the integrated test cohort (containing the validation cohort, the temporal test cohort, and the external test cohort) using the combined model.

### 2.10 Statistical analysis

Data analysis was performed with R software (version 4.2.1). The normality of data distributions was tested using the Kolmogorov- Smirnov test. Skewed data were reported as median (interquartile range) and analyzed using the Mann-Whitney *U* test. Categorical data were presented as frequencies (percentages) and evaluated *via* the chi-square test. Model performance metrics included AUC of ROC curves, accuracy, sensitivity, specificity, positive predictive value, negative predictive value, precision, recall, F1 score, and Brier score. Statistical significance was defined as a p-value < 0.05.

## 3 Results

### 3.1 Baseline characteristics

The training and validation cohort included 271 thyroid lobes from 229 patients (42 with bilateral PTC tumor), consisting of 52 males (22.7 %) and 177 females (77.3 %), with an average age of 46.69 ± 13.68 years. Among these patients, 146 were lymph node metastasis-positive (LNM+), and 125 were negative (LNM-). The temporal test cohort contained 59 lobes from 46 patients, while the external test cohort included 66 lobes from 62 patients. Statistical analysis across all datasets showed no significant differences in clinical variables, imaging features, or laboratory indicators between the training and validation cohort (Table 1).

**Table 1:** Comparison of thyroid lobe characteristics across different cohorts.

Characteristics^a^	Training cohort	Validation cohort	Temporal test cohort	External test cohort	*p*^b^	*p*^c^	*p*^d^
	LNM status	LNM status	LNM status	LNM status			
	yes (n = 102), no (n = 87)	yes (n = 44), no (n = 38)	yes (n = 33), no (n = 26)	yes (n = 32), no (n = 34)			
Age (years)	48.00 (35.00, 57.00)	50.00 (34.25, 58.75)	49.00 (40.00, 55.00)	50.00 (39.25, 55.00)	0.608	0.41	0.228
Gender					0.197	0.658	1.000
Male	50 (26 %)	15 (18 %)	18 (31 %)	17 (26 %)			
Female	139 (74 %)	67 (82 %)	41 (69 %)	49 (74 %)			
BMI	24.73 (22.21, 26.67)	24.98 (22.39, 26.35)	24.77 (23.15, 26.40)	25.01 (24.14, 25.96)	0.952	0.896	0.273
Tumor size group					0.249	0.125	0.021
Small (≤5 mm)	44 (23 %)	20 (24 %)	13 (22 %)	22 (33 %)			
Medium (5–10 mm)	73 (39 %)	39 (48 %)	31 (53 %)	31 (47 %)			
Large (>10 mm)	72 (38 %)	23 (28 %)	15 (25 %)	13 (20 %)			
Multiple nodules					0.892	0.798	0.138
Yes	84 (44 %)	35 (43 %)	28 (47 %)	37 (56 %)			
No	105 (56 %)	47 (57 %)	31 (53 %)	29 (44 %)			
Calcify					0.368	0.430	0.006
Yes	57 (30 %)	30 (37 %)	14 (24 %)	33 (50 %)			
No	132 (70 %)	52 (63 %)	45 (76 %)	33 (50 %)			
Cystic					0.370	0.129	< 0.001
Yes	14 (7 %)	3 (4 %)	1 (2 %)	35 (53 %)			
No	175 (93 %)	79 (96 %)	58 (98 %)	31 (47 %)			
FT3 (pmol/L)	4.76 (4.39, 5.14)	4.75 (4.40, 4.90)	4.71 (4.42, 5.02)	4.74 (4.31, 5.43)	0.262	0.453	0.957
FT4 (pmol/L)	16.40 (14.70, 18.50)	16.40 (14.9, 17.98)	17.70 (14.65, 19.70)	16.85 (14.43, 19.55)	0.959	0.368	0.340
TSH (mIU/L)	1.75 (1.24, 2.70)	1.75 (1.34, 2.53)	2.17 (1.46, 2.72)	1.72 (1.10, 3.12)	0.786	0.055	0.781
TGAb (IU/mL)	16.90 (15.30, 22.20)	16.90 (15.48, 28.10)	16.70 (16.00, 19.00)	17.80 (17.22, 19.10)	0.324	0.450	0.002
TPOAb (IU/mL)	13.00 (12.20, 14.00)	13.00 (13.00, 13.23)	15.65 (15.65, 15.65)	12.60 (9.76, 17.40)	0.695	< 0.001	0.009

Note. A total of 189 thyroid lobes were included in the training cohort, 82 in the validation cohort, 59 in the temporal test cohort, and 66 in the external test cohort. ^a^Biased data are expressed as median (IQR) for continuous variables and as no. (%) for categorical variables. The Mann–Whitney *U* test was applied to compare continuous variables, and the chi-squared test was used for categorical variables. *p*^b^, indicates the significant difference between the training cohort and the validation cohort; *p*^c^, represents the significant difference between the training cohort and the temporal test cohort; *p*^d^, represents the significant difference between the training cohort and the external test cohort. FT3, Free Triiodothyronine; FT4, Free Thyroxine; TSH, Thyroid-Stimulating Hormone; TGAb, Thyroglobulin Antibody; TPOAb, Thyroid Peroxidase Antibody.

### 3.2 Feature selection and construction of DLRad score

**Fig. 3:**
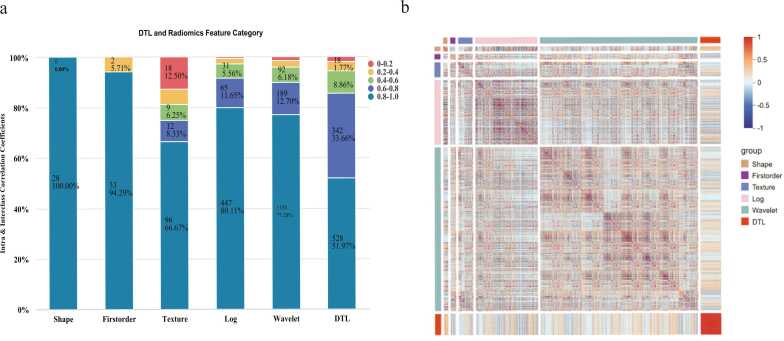
(a) Distribution of Retained Features Based on ICC Values, with features having ICC > 0.8 indicating higher robustness (shaded in blue). (b) Heatmap of features post-ICC filtering (≥ 0.8), showing correlations between feature types.

**Fig. 4:**
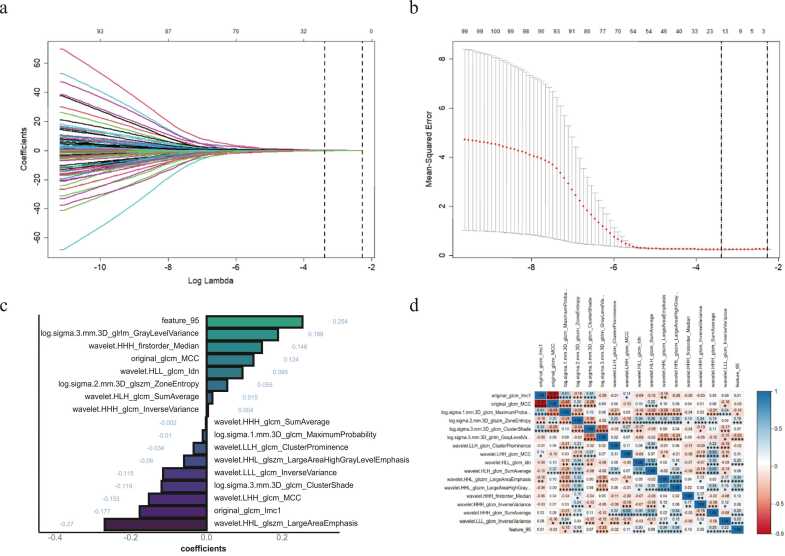
(a) LASSO Feature Selection and Tuning, with the vertical dashed line indicating the optimal penalty coefficient λ for non-zero features; (b) AUC curve plotted *via* 10-fold cross-validation. (c) The 12 features retained after LASSO filtering and their respective weight coefficients; (d) Heatmap of the 12 selected features.

**Fig. 5:**
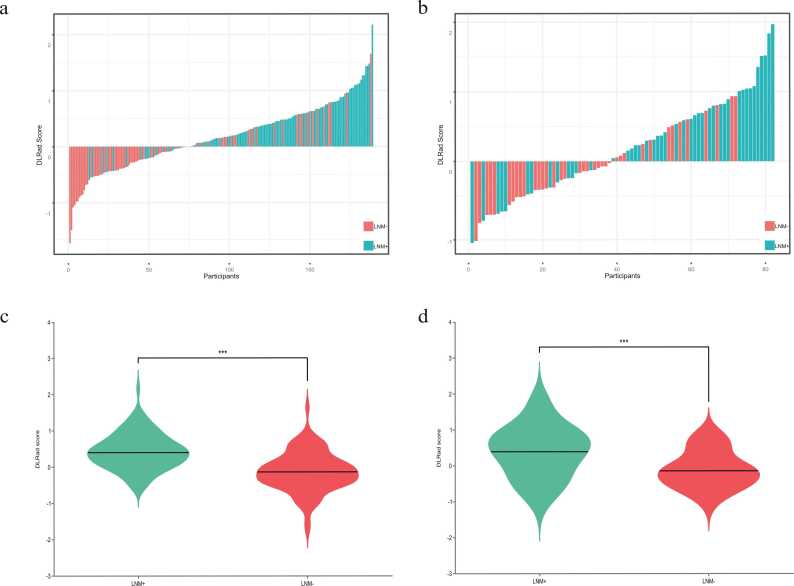
DLRad score waterfall plot for the training cohort (a) and validation cohort (b), with comparative analysis in the training cohort (c) and internal test cohort (d), showing higher scores in the LNM+ group than in the LNM- group, with statistical significance (p < 0.001).

A total of 512 DTL features and 1130 radiomics features were extracted from CT images, resulting in a combined dataset of 1642 features. Feature filtering using intraclass and interclass correlation coefficients reduced this dataset to 1192 features (Fig. 3). Further correlation filtering retained 50 features, with Pearson coefficients above 0.7 indicating high correlation. LASSO regression (λ1 min = 0.03382391) with 10-fold cross-validation ultimately selected 17 predictive features for LNM (Fig. 4), including 1 DTL feature, 2 texture features, 3 LOG features, and 9 wavelet features, as summarized in Table 2. DLRad scores for all lobes were computed using a linear formula. Analysis indicated significant differences (p < 0.001) between LNM-positive and LNM-negative lobes in both the training and validation cohort. For the selected 37 thyroid lobes containing PTC and their contralateral benign counterparts, DLRad scores also showed significant differences (p = 0.002), as illustrated in Fig. 5 and Table 2.

**Table 2:** Features and Z-score parameters selected post-LASSO regression, with differences between benign and malignant thyroid lobes.

Feature name	Weighting coefficient	Average (μ)	Variance (o)	PTC thyroid lobe	Benign thyroid lobe	*p* value
Intercept	0.172122867	-	-	-	-	-
Original_glcm_Imc1	−0.177022396	−0.182831306	0.054646205	0.065 ± 0.195	−0.067 ± 0.149	< 0.001
Original_glcm_MCC	0.123951226	0.618672891	0.093930239	0.044 ± 0.122	−0.05 ± 0.119	< 0.001
Log-sigma−1-mm−3D_glcm_MaximumProbability	−0.010245644	0.479560437	0.064437084	0.003 ± 0.013	−0.005 ± 0.009	0.001
Log-sigma−2-mm−3D_glszm_ZoneEntropy	0.054690506	5.211098628	0.360383953	0.014 ± 0.049	0.004 ± 0.048	0.366
Log-sigma−3.mm−3D_glcm_ClusterShade	−0.11916376	−71.91888711	35.52190946	0.018 ± 0.153	−0.013 ± 0.103	0.123
Log-sigma−3-mm−3D_glrlm_GrayLevelVariance	0.188991786	6.204187979	1.21207322	−0.024 (−0.163, 0.071)	−0.045 (−0.136, 0.117)	0.197
Wavelet-LLH_glcm_ClusterProminence	−0.034367001	336.9299999	3065.514353	0.004 (0.003, 0.004)	0.004 (0.004, 0.004)	0.042
Wavelet-LHH_glcm_MCC	−0.15272924	0.375843472	0.136389357	0.017 ± 0.128	−0.011 ± 0.135	0.202
Wavelet-HLL_glcm_Idn	0.094992867	0.96315397	0.013994124	0.017 (−0.092, 0.106)	0.005 (−0.068, 0.050)	0.541
Wavelet-HLH_glcm_SumAverage	0.015310386	7.698680862	3.08366493	−0.003 (−0.013, 0.011)	−0.004 (−0.013, 0.006)	0.163
Wavelet-HHL_glszm_LargeAreaEmphasis	−0.269669339	1,049,574.863	3,063,385.348	0.060 (0.007, 0.072)	0.070 (0.017, 0.082)	0.338
Wavelet-HHL_glszm_LargeAreaHighGrayLevelEmphasis	−0.059642513	22,706,356.23	98,916,165.41	0.009 (0.002, 0.012)	0.011 (0.006, 0.012)	0.029
Wavelet-HHH_firstorder_Median	0.146424596	0.002059445	0.054479135	0.052 ± 0.122	0.025 ± 0.144	0.406
Wavelet-HHH_glcm_InverseVariance	0.0037	0.510835856	0.002381123	0.001 ± 0.002	−0.001 ± 0.002	0.004
Wavelet-HHH_glcm_SumAverage	−0.002081309	6.850438518	2.205983292	0.000 (−0.006, 0.001)	0.000 (0.000, 0.002)	0.096
Wavelet-LLL_glcm_InverseVariance	−0.11516717	0.446475401	0.023038523	0.041 (−0.082, 0.105)	−0.031 (−0.087, 0.033)	0.105
Feature_95	0.253537381	0.293081067	0.022055408	−0.081 (−0.165, 0.120)	−0.024 (−0.203, 0.147)	0.689
aDLRad Score	-	-	-	0.380 ± 0.673	0.009 ± 0.583	0.002

a The DLRad score, calculated through linear weighting, was significantly higher in the 37 included PTC thyroid lobes compared to their contralateral benign thyroid lobes.

### 3.3 Model construction and evaluation

**Fig. 6:**
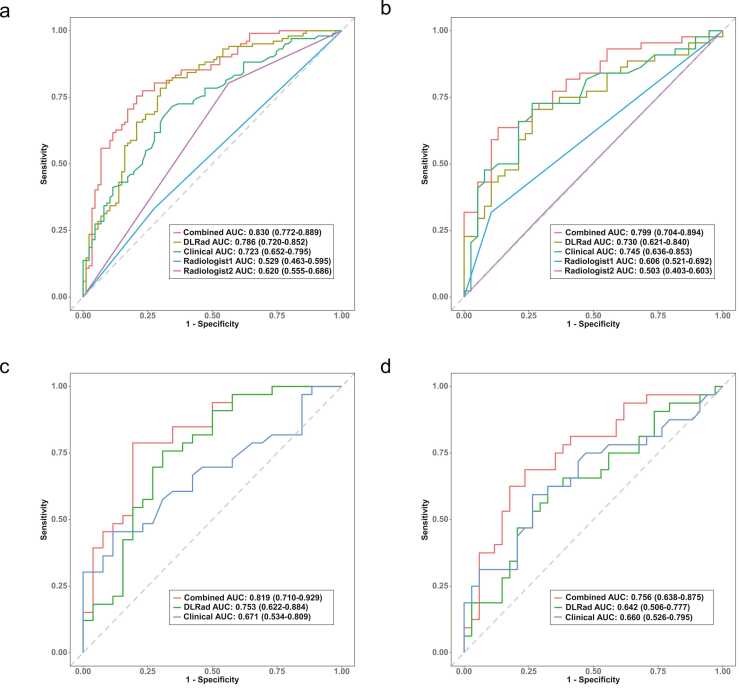
ROC curves comparing multiple models in the training cohort (a), validation cohort (b), temporal test cohort (c), and external test cohort (d), with the combined model exhibiting higher AUC values.

**Fig. 7:**
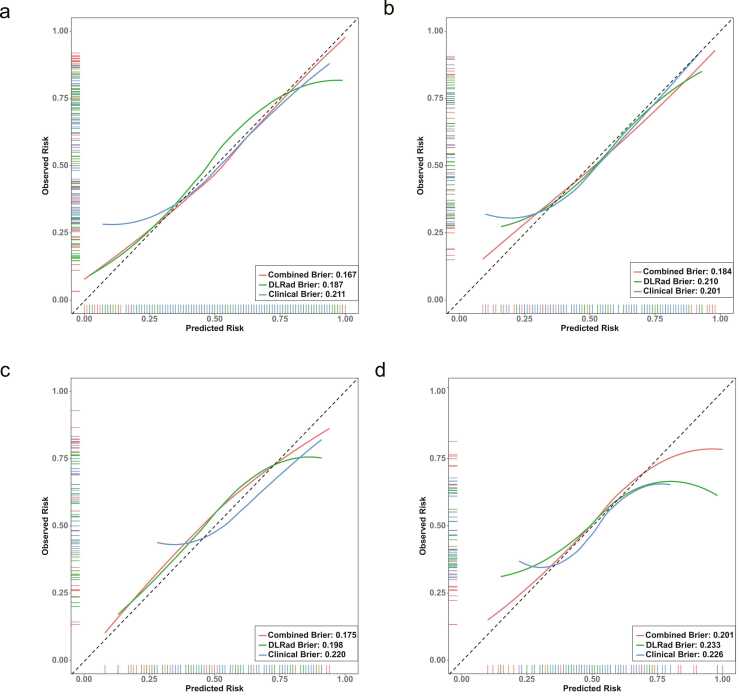
Calibration curves for multiple models in the training cohort (a), validation cohort (b), temporal test cohort (c), and external test cohort (d).

**Fig. 8:**
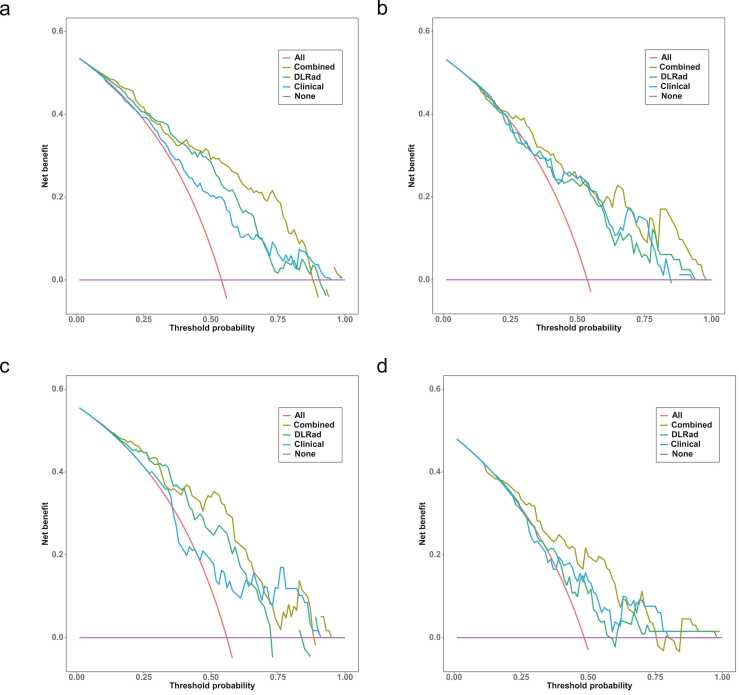
Decision curve analysis for multiple models in the training cohort (a), validation cohort (b), temporal test cohort (c), and external test cohort (d).

Univariate and multivariate logistic regression analyses identified independent LNM risk factors in the training cohort (Table 3). Clinical, DLRad, and combined models were developed based on Age, Gender, Multiple nodules and Tumor size group across all datasets. The combined model achieved AUCs of 0.830, 0.799, 0.819, and 0.756 in the training, validation, temporal test, and external test cohort, respectively, outperforming both the clinical model (AUCs: 0.723, 0.745, 0.671, 0.660) and the DLRad model (AUCs: 0.786, 0.730, 0.753, 0.642) (Table 4 and Fig. 6). Calibration curves and Brier scores indicated that the combined model demonstrated higher accuracy across all datasets (Fig. 7). DCA showed that for threshold probabilities above 0.2, the combined model provided a net benefit and greater clinical advantages across all data cohorts (Fig. 8). The findings of the performance evaluation of the different size subgroups utilising the combined model demonstrated that for larger tumors, the AUCs in the training cohort and the integrated test cohort were 0.858 and 0.810, respectively, while for smaller tumors, the corresponding AUCs were 0.784 and 0.767, respectively (Table 5).

**Table 3:** Results of univariate and multivariate logistic regression analyses.

characteristics	Univariate analysis			Multivariate analysis (clinical model parameters)			Multivariate analysis (combined model parameters)		
	*β* coefficient	*OR* (95 %*CI*)	*p* value	*β* coefficient	*OR* (95 %*CI*)	*p* value	*β* coefficient	*OR* (95 %*CI*)	*p* value
Age	−0.049	0.952 (0.928–0.975)	< 0.001	−0.060	0.941 (0.914–0.967)	< 0.001	−0.056	0.946 (0.914–0.975)	0.001
Gender	0.919	2.507 (1.277–5.116)	0.009	0.720	2.055 (0.982–4.434)	0.060	0.439	1.551 (0.699–3.504)	0.283
BMI	0.001	1.001 (0.925–1.083)	0.983	-	-	-	-	-	-
Cystic	−0.806	0.447 (0.133–1.347)	0.163	-	-	-	-	-	-
Calcify	0.539	1.714 (0.913–3.279)	0.097	-	-	-	-	-	-
Tumor size group	0.509	1.663 (1.140–2.456)	0.009	0.621	1.860 (1.226–2.870)	0.004	0.351	1.421 (0.880–2.306)	0.150
Multiple nodules	0.670	1.954 (1.092–3.535)	0.025	1.088	2.969 (1.519–6.022)	0.002	1.120	3.065 (1.449–6.806)	0.004
FT3	0.444	1.560 (0.991–2.513)	0.060	-	-	-	-	-	-
FT4	0.062	1.064 (0.957–1.185)	0.255	-	-	-	-	-	-
TSH	0.081	1.084 (0.913–1.332)	0.384	-	-	-	-	-	-
TPOAb	0.000	1.000 (1.000–1.001)	0.289	-	-	-	-	-	-
TGAb	−0.002	0.998 (0.995–1.002)	0.361	-	-	-	-	-	-
DLRad Score	2.237	9.366 (4.616–20.890)	< 0.001	-	-	-	2.015	7.503 (3.535–17.480)	< 0.001

Note. OR, odds ratios; CI, confidence interval; FT3, Free Triiodothyronine; FT4, Free Thyroxine; TSH, Thyroid-Stimulating Hormone; TGAb, Thyroglobulin Antibody; TPOAb, Thyroid Peroxidase Antibody.

**Table 4:** Predictive performance of multiple models across different cohorts.

Models	AUC (95 %CI)	Accuracy	Sensitivity	Specificity	PPV	NPV	Precision	Recall	F1	Brier
Training cohort (n = 189)										
DLRad model	0.786 (0.720–0.852)	0.746	0.784	0.701	0.755	0.735	0.755	0.784	0.769	0.187
Clinical model	0.723 (0.652–0.795)	0.688	0.716	0.655	0.709	0.663	0.709	0.712	0.712	0.211
Combined model	0.830 (0.772–0.889)	0.773	0.755	0.793	0.811	0.734	0.811	0.755	0.782	0.167
Radiologist1	0.529 (0.463–0.595)	0.513	0.333	0.724	0.586	0.481	0.586	0.333	0.425	0.248
Radiologist2	0.620 (0.555–0.686)	0.635	0.804	0.437	0.626	0.655	0.626	0.804	0.704	0.232
Validation cohort (n = 82)										
DLRad model	0.730 (0.621–0.840)	0.720	0.705	0.737	0.756	0.683	0.756	0.705	0.729	0.210
Clinical model	0.745 (0.636–0.853)	0.732	0.727	0.737	0.762	0.700	0.762	0.727	0.744	0.201
Combined model	0.799 (0.653–0.863)	0.744	0.634	0.868	0.849	0.674	0.849	0.636	0.727	0.184
Radiologist1	0.606 (0.521–0.692)	0.585	0.318	0.895	0.779	0.531	0.778	0.318	0.452	0.242
Radiologist2	0.503 (0.403–0.603)	0.512	0.705	0.290	0.535	0.458	0.535	0.705	0.608	0.266
Temporal test cohort (n = 59)										
DLRad model	0.753 (0.622–0.884)	0.729	0.758	0.692	0.758	0.692	0.758	0.758	0.758	0.198
Clinical model	0.671 (0.534–0.809)	0.644	0.455	0.885	0.833	0.561	0.833	0.455	0.588	0.220
Combined model	0.819 (0.710–0.929)	0.797	0.788	0.808	0.839	0.750	0.839	0.788	0.813	0.175
External test cohort (n = 66)										
DLRad model	0.642 (0.506–0.777)	0.652	0.625	0.677	0.645	0.657	0.645	0.625	0.635	0.233
Clinical model	0.660 (0.526–0.795)	0.667	0.594	0.735	0.679	0.658	0.679	0.594	0.633	0.226
Combined model	0.756 (0.638–0.875)	0.727	0.688	0.765	0.733	0.722	0.733	0.688	0.710	0.201

Note. The combined model is a fusion of the DLRad model and the clinical model; AUC, area under the curve; NPV, negative predictive value; PPV, positive predictive value.

**Table 5:** Performance evaluation of the combined model for LNM in subgroups of different sized PTCs.

	AUC (95 %CI)	Accuracy	Sensitivity	Specificity
Training cohort: size ≤ 5 mm	0.784 (0.635–0.932)	0.796	0.800	0.793
Training cohort: size 5–10 mm	0.834 (0.744–0.924)	0.781	0.698	0.900
Training cohort: size > 10 mm	0.858 (0.765–0.951)	0.819	0.841	0.786
Integrated test cohort: size ≤ 5 mm	0.767 (0.641–0.893)	0.746	0.556	0.927
Integrated test cohort: size 5–10 mm	0.796 (0.709–0.882)	0.753	0.729	0.774
Integrated test cohort: size > 10 mm	0.810 (0.673–0.947)	0.804	0.853	0.706

Note: integrated test cohort, validation cohort & temporal test cohort & external test cohort; LNM, lymph node metastasis; PTC, papillary thyroid cancer.

### 3.4 Model comparison

**Fig. 9:**
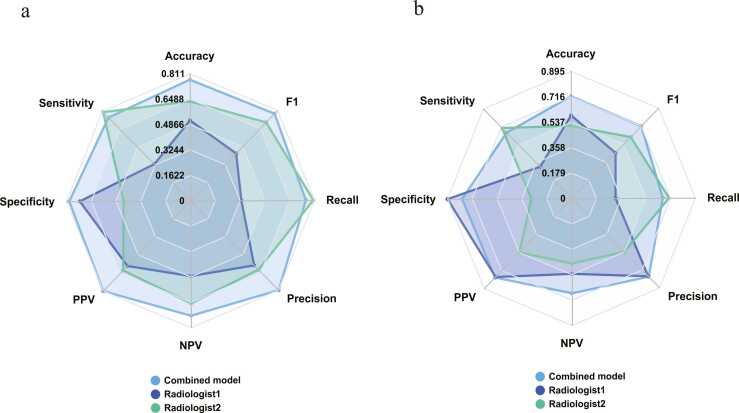
Comparison of the combined model and radiologists in the training cohort (a) and validation cohort (b).

**Fig. 10:**
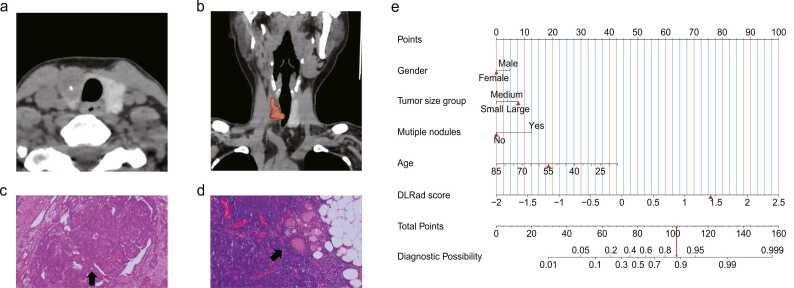
A 55-year-old female patient presented with a single nodule in the right thyroid lobe with a maximum size of 20 mm. The segmentation of this thyroid lobe is shown in transverse (a) and coronal (b) views. Postoperative pathological examination confirmed the diagnosis of PTC (c) with lateral zone LNM (d). The risk of developing LNM in this patient's right-sided PTC was predicted using a nomogram, and the scores for all variables were summed to give a total score of 102, corresponding to a predicted risk probability of 88 % (e).

The DeLong test demonstrated that the combined model significantly outperformed the two human imaging experts. In the training cohort, the combined model exhibited notable superiority over the other models (Table 6 and Fig. 9), with statistically significant differences (p < 0.05, p < 0.001). However, no significant differences were observed among the models in the validation, temporal test, and external test cohorts. A nomogram was developed to visually represent the predictions of the combined model (Fig. 10).

**Table 6:** DeLong test comparing AUC values between different models and human radiologists.

		Combined vs DLRad	Combined vs Clinical	Combined vs Rad. 1	Combined vs Rad. 2	DLRad vs Clinical
Training cohort	*Z*	2.180	3.293	6.738	5.029	1.303
	*p*	0.029	< 0.001	< 0.001	< 0.001	0.193
Validation cohort	*Z*	1.712	1.526	2.903	3.979	0.206
	*p*	0.087	0.127	0.003	< 0.001	0.836
Temporal test cohort	*Z*	1.752	2.339	-	-	0.874
	*p*	0.080	0.019	-	-	0.388
External test cohort	*Z*	1.712	1.735	-	-	0.182
	*p*	0.087	0.083	-	-	0.856

Note. Combined, DLRad + Clinical; Rad., Radiologist.

## 4 Discussion

In this retrospective study, a combined model incorporating DTL features, radiomic features, and clinical variables from CT images was developed to predict LNM risk in patients with PTC. The model demonstrated superior predictive performance compared to human imaging experts, with a nomogram that further facilitated clinical decision-making. Strong performance in both temporal and external test cohort underscored the model's generalizability, offering clinicians a valuable tool for preoperative LNM assessment in patients with PTC, supporting personalized treatment planning.

Preoperative LNM evaluation is essential for PTC management; however, US and CT often struggle to accurately assess small or early-stage lymph nodes [31]. This limitation has led some surgeons to conduct prophylactic cervical lymph node dissection, though the clinical benefits of this approach are debated due to risks of complications that may adversely impact quality of life and recovery [32], [33]. Additionally, LNM correlates with higher local recurrence rates and poorer prognosis [34], underscoring the need for effective preoperative predictive techniques. With recent advances in artificial intelligence (AI), DTL and radiomics have gained traction in clinical studies. DTL leverages pre-trained models that can be fine-tuned for new datasets, achieving strong performance with reduced training times, even on smaller datasets [35]. This study employed a 3D ResNet-18 convolutional neural network to extract deep learning features, utilizing residual blocks to address vanishing gradients in deep network training. The 3D convolution layers enabled comprehensive spatial information capture [36]. Radiomics, through feature extraction algorithms, quantifies aspects of medical images, including first-order, texture, Laplacian, and wavelet features, and is widely applied in disease prediction and assessment research [37]. The fusion of DTL and radiomic features provided spatial and quantitative complementarity, enhancing both model generalizability and predictive accuracy.

Previous studies have demonstrated that AI methods based on CT imaging can predict LNM risk in PTC. For instance, Lee et al. [38] developed a deep learning CAD system that achieved an AUC of 0.953 for cervical LNM prediction. Masuda et al. [39] found that machine learning approaches incorporating texture analysis (AUC 0.86) significantly outperformed traditional morphological methods (AUC 0.43–0.54). Li et al. [40] constructed a model combining radiomic features with clinical risk factors, achieving an AUC of 0.764 in external validation. In our study, a combined model of DTL features, radiomic features, and clinical variables significantly outperformed human experts (AUC 0.830–0.756 vs. 0.620–0.497). Feature selection retained 1 DTL feature, 2 texture features, 2 Gaussian features, and 7 wavelet features. The DTL feature captured high-dimensional information; texture features represented local gray-level distributions and spatial structures; Gaussian features extracted edge and local structural details; and wavelet features enabled multi-scale and multi-directional analysis [41]. Together, these features offered a multi-dimensional perspective on the differences between thyroid lobes containing PTC with and without LNM.

Unlike previous studies, this study was based on images from NCCT, which is still the most commonly used initial examination modality in clinical practice, despite the advantages of CECT in assessing PTC invasion of surrounding structures, which can better show the relationship of the tumor to organs and major vessels. We intentionally employed NCCT for both radiomic modeling and radiologists’ lymph node assessment for three reasons: First, thyroid tissue is highly sensitive to ionizing radiation, a known risk factor for PTC [42]. NCCT requires fewer scans than CECT, significantly reducing radiation exposure. Second, NCCT avoids iodine-based contrast agents, thereby minimizing risks of thyroid dysfunction and interference with subsequent I-131 therapy [43]. Third, our study aimed to establish a fair human-machine comparison framework. Since the predictive model was developed using NCCT, radiologists’ assessments were also performed on NCCT to eliminate modality-related bias. This approach ensured direct comparability between human and machine performance. Additionally, NCCT is frequently utilized in clinical settings, with incidental thyroid nodules often detected during routine chest CT exams. Therefore, focusing on NCCT features also promotes research into opportunistic screening [44].

Traditional studies on predicting LNM have primarily focused on the relationship between lymph nodes and PTC tumor [45], [46]. However, the large number of neck lymph nodes and limited specificity of CT and US in identifying certain metastatic lymph nodes [47] complicate the alignment of imaging-detected lymph nodes with pathological findings. Additionally, the localization of PTC tumor can be subject to human bias, particularly in cases with multiple nodules. Delineating nodule boundaries, especially with contrast enhancement, may also vary due to human interpretation. This study employed a semi-lobar segmentation approach for thyroid tissue to provide a more comprehensive assessment of overall characteristics, minimizing biases associated with single-nodule segmentation and reducing potential confounding from full thyroid segmentation [48]. Subgroup analysis of tumor size subgroups shows that, although the combined model has a slightly lower AUC in smaller tumors compared to larger tumors, it remains effective in extracting feature information from the half lobe of the thyroid that is significant for LNM. The model’s performance is consistent across different size subgroups in both the training and integrated test cohort, further demonstrating the generalizability of the segmentation scheme. Moreover, a comparison between unilateral PTC lobes and contralateral benign lobes revealed significantly higher DLRad scores in PTC lobes, indicating that model features better represent PTC-specific information. The lower DLRad scores observed in benign lobes indirectly suggest a lower likelihood of false positives in benign lobes, enhancing the model’s clinical utility.

Kim et al. [49] identified younger age, male gender, multifocality, and large tumor size as independent risk factors for LNM, aligning with our findings. Univariate and multivariate logistic regression analysis showed that younger age (OR: 0.946, 95 % CI: 0.914–0.975), male gender (OR: 1.551, 95 % CI: 0.699–3.504), multiple nodules (OR: 3.065, 95 % CI: 1.449–6.806) and tumor size group (OR: 1.421, 95 % CI: 0.880–2.306) were significantly correlated with LNM. Younger patients are often associated with more aggressive tumor growth, a more active lymphatic system, and a stronger immune response. Male patients tend to present more aggressive PTC, which may be linked to androgen levels and higher rates of BRAF gene mutations. Multifocality increases tumor burden, raising the likelihood of lymphatic spread. These factors likely interact, collectively elevating LNM risk [2]. Although some studies have identified lymph node enlargement and calcification as LNM indicators [50], further research is required to explore the associations between tumor calcification, cystic degeneration, and LNM.

This study has several limitations. Firstly, as a retrospective analysis, it only included lobe data with confirmed PTC pathology. In clinical practice, alternative diagnostic methods are necessary to confirm the pathological type of thyroid nodules prior to applying this model, which may add diagnostic complexity and increase time costs, potentially limiting the model's immediate applicability. Secondly, in bilateral PTC tumors, the study divided thyroid tissue into left and right lobes for feature extraction. While this segmentation approach allowed for a focused examination of specific lobe areas and accounted for the cervical lymphatic network's characteristics, including cross-regional and contralateral LNM risks in PTC [51], [52], it may increase the model’s false-positive rate, introducing potential bias. Despite reducing bias in unilateral lobes, this strategy could affect the model's accuracy. Additionally, the study did not exclude cases of Hashimoto’s thyroiditis or granulomatous inflammation, which may reduce NCCT contrast in thyroid tissue and complicate manual segmentation. Future research will incorporate diagnostic models for both benign and malignant lobes to expand clinical applicability and utility. Furthermore, combining multimodality such as CECT and exploring the use of varied deep transfer learning networks for feature extraction could enhance the model’s accuracy in predicting LNM, increasing its potential for clinical integration.

## 5 Conclusion

In conclusion, this study introduces a combined model and nomogram that integrates deep transfer learning, radiomics, and clinical variables from NCCT imaging with half lobe segmentation of the thyroid. The model exhibits moderate to good performance in predicting LNM in patients with PTC, supporting the development of personalized treatment plans and enhancing clinical decision-making.

## Ethical approval

This study was approved by the Ethics Committee of the Fourth Affiliated Hospital of Nanjing Medical University (Approval No. 20240628-K077). Due to the retrospective nature of the study, requirements for informed consent were waived.

## Funding

This study was supported by funding from the General project of the Science and Technology Development Fund of Nanjing Medical University (NMUB20230037).

## CRediT authorship contribution statement

**Zeng Hanling:** Methodology. **Tang Wuliang:** Data curation. **He Jian:** Project administration. **Tao Jing:** Supervision. **Bai Zhuojie:** Methodology. **Wang You:** Validation. **Wu Di:** Validation. **Wang Hao:** Writing – original draft, Software, Resources, Methodology, Data curation. **Du Yusheng:** Data curation. **Wang Xuan:** Writing – review & editing.

## Declaration of Generative AI and AI-assisted technologies in the writing process

During the preparation of this work the author(s) used Chatgpt 4o in order to translation. After using this tool, the author(s) reviewed and edited the content as needed and take(s) full responsibility for the content of the publication.

## Declaration of Competing Interest

The authors declare that they have no known competing financial interests or personal relationships that could have appeared to influence the work reported in this paper.

## Data Availability

The data utilised and/or analysed during the present study are available from the corresponding author upon reasonable request.
